# Apoptotic cell administration is detrimental in murine renal ischaemia reperfusion injury

**DOI:** 10.1186/s12950-014-0031-6

**Published:** 2014-10-10

**Authors:** Emily E Hesketh, David C Kluth, Jeremy Hughes

**Affiliations:** MRC Centre for Inflammation Research, Queen’s Medical Research Institute, University of Edinburgh, 47 Little France Crescent, Edinburgh, EH16 4TJ UK

**Keywords:** Ischaemia reperfusion injury, Acute kidney injury, Apoptotic cells, Inflammation, Microvasculature

## Abstract

**Background:**

Acute kidney injury induced by renal ischaemia reperfusion injury (IRI) is characterised by renal failure, acute tubular necrosis (ATN), inflammation and microvascular congestion. The administration of apoptotic cells (ACs) has been shown to reduce inflammation in various organs including the liver and kidney. This study explored whether AC administration prior to the induction of renal IRI was protective.

**Findings:**

Renal IRI was induced in Balb/c mice by clamping the renal blood vessels for either 20, 24 or 25 minutes to induce mild, moderate or severe kidney dysfunction respectively. Renal function and injury was determined 24 hours following IRI by measurement of plasma creatinine and ATN scoring respectively. ACs were generated from Balb/c thymocytes and classified as either predominantly early or late apoptotic by Annexin-V and propidium iodide staining. Early AC administration prior to severe IRI had no influence on plasma creatinine or ATN severity. In contrast, administration of early or late ACs significantly worsened renal function in mice with mild or moderate renal IRI, respectively, compared to PBS treated controls, though ATN scores were comparable. Despite ACs exerting pro-coagulant effects, the worsening of renal function was not secondary to increased microvascular congestion, inferred by fibrin and platelet (CD41) deposition, or inflammation, assessed by neutrophil infiltration.

**Conclusions:**

Despite the AC-derived protection demonstrated in other organs, ACs do not protect mice from renal IRI. ACs may in fact further impair renal function depending on injury severity. These data suggest that AC-derived protection is not translationally relevant for patients with acute kidney injury induced by ischaemic injury.

**Electronic supplementary material:**

The online version of this article (doi:10.1186/s12950-014-0031-6) contains supplementary material, which is available to authorized users.

## Findings

### Introduction

Acute kidney injury (AKI) induced by renal ischaemia reperfusion injury (IRI) is a significant risk factor for patients undergoing renal transplantation or any major surgery that may transiently reduce renal blood flow. Ischaemic AKI has a complex pathogenesis involving acute inflammatory responses, endothelial and tubular cell injury that determine injury severity [1-3]. Inflammatory responses lead to further endothelial dysfunction and injury with increased leukocyte infiltration and reduced microvascular blood flow [2]. Pro-inflammatory cytokines such as tumour necrosis factor-α (TNF-α) and interleukin (IL)-1β are released by infiltrating leukocytes and injured tubular epithelial cells which act to potentiate inflammation and associated injury [4,5]. As inflammation plays a key role following renal IRI it is not surprising that the resultant injury may be attenuated by anti-inflammatory treatments [6]. There is an urgent need for novel treatments for AKI that reduce inflammation as current clinical treatments are purely supportive with no specific therapy available.

Increased numbers of apoptotic cells (ACs) are found at sites of inflammation. Surface exposure of phosphatidylserine (PS) on the AC surface promotes AC clearance by macrophages [7] which facilitates the resolution of inflammation [8]. Macrophages that ingest ACs adopt an anti-inflammatory phenotype with secretion of transforming growth factor-β (TGF-β) and IL-10 [9]. Exogenous AC administration reduces inflammation in various experimental models including lipopolysaccharide (LPS)-induced shock [10], collagen induced arthritis (CIA) [11] as well as lung [12] and liver inflammation [13]. The anti-inflammatory mechanisms of exogenous AC administration are diverse and include (i) the induction of IL-10 production by Kupffer cells by TGF-β bound to the AC surface [13], (ii) the induction of IL-10 producing CD19^+^ regulatory B cells that stimulate a population of IL-10 secreting antigen-specific CD4^+^ T cells in CIA [11] and (iii) binding of LPS to ACs accompanied by reduced serum TNF-α levels and increased serum IL-10 levels in LPS shock [10]. Ren *et al.* also observed suppressed neutrophil infiltration in the kidney following AC administration in LPS-induced shock [10]. In addition, it has been shown that both apoptotic and necrotic cells exert anti-inflammatory effects [14] suggesting that these effects are not confined to intact ACs.

In view of these previous studies AC administration may represent a novel pretreatment for AKI secondary to renal IRI and act to limit the resultant inflammation and tissue injury. This short study explored whether ACs administered 24 hr prior to the induction of renal IRI could protect Balb/c mice from functional and structural renal injury. The findings contrast with the AC-derived protection observed in other organs and suggest that AC administration is either neutral or, depending upon the severity of the ischaemic injury, may act to worsen renal function.

### Methods

#### Mice

Experiments were performed on male Balb/c mice aged between 4–8 weeks (Harlan). All animal procedures were performed under a Project License in accordance with guidelines set out by the United Kingdom’s Home Office under the Animal (Scientific Procedures) Act of 1986 and the University of Edinburgh’s Biological Services Department.

#### Preparation and administration of viable and apoptotic thymocytes

Dissociated thymocytes harvested from the thymi of Balb/c mice aged 4-weeks were used fresh or incubated for 20 hr in RPMI 1640 (PAA Laboratories) or RMPI 1640 supplemented with 1 μM dexamethasone (Oragon). Cell viability was assessed by Annexin-V (BioLegend) and Propidium Iodide (PI; Invitrogen) staining assessed by flow cytometry on a BD Calibur cytometer. ACs were classified as either early (Annexin-V^+^ PI^-^) or late (Annexin-V^+^ PI^+^) apoptotic. Either PBS (control) or 20×10^6^ viable thymocytes or ACs was administered intravenously to mice 24 hr prior to renal IRI.

#### Thymocyte phenotyping

Fresh thymocytes were prepared as described and stained with the following anti-mouse antibodies: PE CD4 (1:200, Clone: RM4-5, BD Pharmingen), APC CD8α (1:200, Clone: 53-6.7, eBioscience), PerCp-Cy5.5 CD11b (1:200, Clone: M1/70, eBiosciences) and Pacific Blue B220 (1:200, Clone: RA3-6B2, BD Pharmingen) before analysis by flow cytometry on a BD LSR Fortessa. Isotype controls were used to determine staining positivity.

#### Renal IRI and assessment of renal function and acute tubular necrosis (ATN)

Detailed methodology is described in Hesketh *et al.*, [15]. In brief male 8-week Balb/c mice underwent a contralateral right nephrectomy before the renal pedicle was clamped with an atraumatic clamp for 22 (mild), 24 (moderate) or 25 minutes (severe injury). Body temperature was maintained at 37°C by a homeostatically controlled blanket (Harvard Apparatus, Boston MA) during the ischaemic period. The peritoneum was then sutured and the skin closed with metallic clips and the anaesthesia reversed. Sham mice underwent a laparotomy with manipulation of the left and right renal pedicle only. Tissue and blood was collected 24 hr post-surgery under terminal anaesthesia. Creatinine concentration [μmol/L] was assessed in plasma samples using a creatininase based method on a Cobas Fara Centrifugal Analyser (Roche Diagnostics, UK) [15]. To determine the ATN score the number of viable (intact cell membrane) and necrotic tubules (compromised cell membrane) were marked and counted in deparaffinised 4 μm kidney sections stained with H&E [15]. The number of necrotic tubules was expressed as a percentage of the total number of tubules (necrotic tubules %). ATN was assessed in 5 images taken at ×200 magnification of the outer stripe of the outer medulla (OSOM) per mouse. ATN was scored in a blinded manner.

#### Immunohistochemistry (IHC)

Deparaffinised or frozen kidney sections, fixed in 70% methanol, cut at a thickness of 4 μM were prepared. Endogenous peroxidase activity was blocked using 0.3% H_2_O_2_ diluted in methanol. Endogenous avidin and biotin activity was blocked using an Avidin/Biotin Blocking Kit (Vector Laboratories). Non-specific binding was blocked using protein block serum-free ready-to-use (Dako). Primary antibodies used were rat anti-mouse CD41 (1:250, Clone: MWReg30, AbDserotec) and rat anti-mouse Gr1 (1:300, Clone: RB6-8C5, BioLegend). A biotinylated secondary rabbit anti-rat IgG (Vector Laboratories) was applied (1:300). R.T.U Vectastain Kit Elite Reagent (Vector Laboratories) was applied before sections were exposed to Dako Liquid DAB + Substrate Chromogen System. Sections were counterstained with Haematoxylin. Frozen sections were mounted with ProLong Gold Antifade Reagent (Life Technologies) and paraffin sections were dehydrated and mounted with Pertex. Negative controls without the primary antibody and control isotype antibodies were included (Additional file 1).

#### Fibrin direct immunofluorescence (IF)

Frozen kidney sections cut at a thickness of 4 μM were fixed with 4% PFA 2% Acetic acid before incubation with rabbit serum (Sigma). Polyclonal Rabbit Anti-Human Fibrinogen/FITC (1:200, Dako Cytomation) which cross-reacts with mouse fibrin was applied before sections were stained with DAPI (Life Technologies). Sections were mounted with ProLong Gold Antifade Reagent (Life Technologies). Positive control tissue was included.

#### Quantification of IF and IHC staining

For each stained section 5 images of the OSOM were taken and positive staining was selected using ImageJ (NIH). The number of positive pixels was expressed as a percentage of the total number of pixels [16]. Sections were examined using either a Zeiss Axioskop 2mot + and Hamamatsu Orca-ER camera or a Zeiss Axioskop microscope and QImaging Micropublisher 3.3 RTV camera. Quantification was performed in a blinded manner.

#### Statistics

Data are expressed as mean ± SEM. Significance was assessed by using unpaired students *t* test, one- or two-way ANOVA where appropriate using Prism software (Graphpad). P values <0.05 were considered significant.

### Results

**Figure 1:**
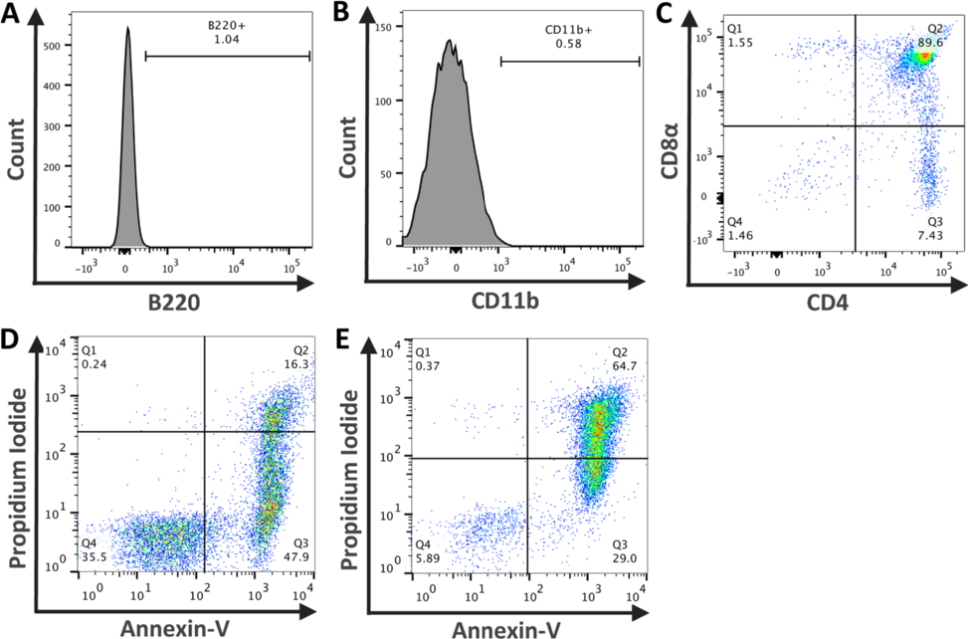
**Representative data illustrating the phenotype of thymocytes and classification of early and late ACs.** Freshly isolated thymocytes were stained with CD11b, B220, CD4 and CD8α and analysed by flow cytometry to assess the phenotype of the cells used to generate ACs. Minimal staining for B220 **(A)** and CD11b **(B)** was found. Approximately 98% of cells gated to exclude debris are lymphocytes and either CD4^+^, CD8α^+^ or CD4^+^CD8α^+^ **(C)**. Prior to administration of ACs cell viability was assessed by Annexin-V and Propidium Iodide (PI) staining and flow cytometry. Overnight culture alone induced predominantly early ACs (47% Annexin-V^+^ PI^-^) **(D)** whilst the addition of 1 μM dexamethasone elicited a population of late ACs (64.7% Annexin-V^+^ PI^+^) **(E)**. Data representative of all AC preparations.

The phenotype of the fresh thymocytes used to generate the ACs was examined by assessing the expression of CD4, CD8, B220 (B cell marker) and CD11b (myeloid cell marker). Minimal expression of B220 (Figure 1A) and CD11b (Figure 1B) confirmed that cell preparations consisted predominantly of thymocytes, 98% of which were either CD4^+^, CD8α^+^ or CD4^+^CD8α^+^ (Figure 1C).

**Figure 2:**
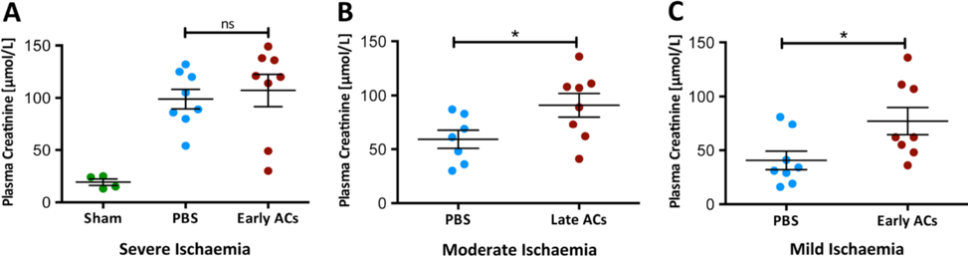
**AC administration prior to moderate and mild renal IRI impaired renal function.** Renal IRI was induced in 8-week old male Balb/c mice by a right nephrectomy and ischaemia induced by occlusion of the left renal pedicle. Sham mice underwent a laparotomy only. Either PBS or 20×10^6^ early or late ACs were administered 24 hr prior to mild (20 mins ischaemia), moderate (24 mins) or severe (25 mins) ischaemic injury. Mice were sacrificed 24 hr following IRI. **(A)** Administration of early ACs did not preserve renal function from severe IRI. **(B)** In contrast, administration of late ACs significantly worsened renal function in mice with moderate IRI. **(C)** Similarly, early ACs administered prior to mild ischaemia significantly worsened renal function. Analysed by one-way ANOVA or students *t*-test * = P ≤ 0.05, ns = non-significant. Sham (*n* = 4), PBS (*n* = 7–8), AC (*n* = 8).

**Figure 3:**
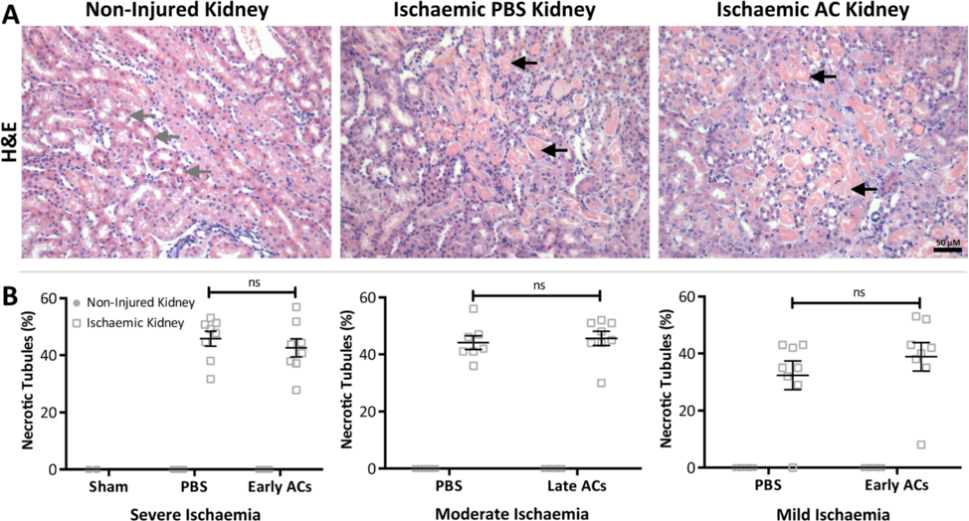
**Acute tubular necrosis (ATN) following ischaemia was unaffected following AC administration.** Renal IRI was induced in 8-week old male Balb/c mice by a right nephrectomy and ischaemia induced by occluding the left renal pedicle for 20 (mild ischaemia), 24 (moderate) or 25 mins (severe). Sham mice underwent a laparotomy only. Either PBS or 20×10^6^ early or late ACs were administered 24 hr prior to renal IRI. Mice were sacrificed 24 hr following IRI. **(A)** Representative images of the outer stripe of the outer medulla (OSOM) in non-injured and ischaemic kidney sections stained with H&E. Tubules within the OSOM were classified as necrotic (black arrow) or healthy (grey arrow) according to cell morphology and integrity. (Magnification: ×200; Scale Bar: 50 μM) **(B)** Scoring of ATN (expressed as the percentage of necrotic tubules) demonstrates that the structural injury remained similar between PBS and AC treated mice with severe, moderate and mild ischaemic injury. Grey circle symbol = Non-injured kidney Grey square symbol = Ischaemic kidney. Data expressed as mean ± SEM and analysed by two-way ANOVA. ns = non-significant. Sham (*n* = 4), PBS (*n* = 7–8), AC (*n* = 8).

To explore the effects of ACs upon renal IRI either PBS or 20×10^6^ predominantly early ACs (Annexin-V^+^ PI^-^, Figure 1D) or predominantly late ACs (Annexin-V^+^ PI^+^, Figure 1E) were administered intravenously to mice 24 hr before renal IRI. Mice were then sacrificed 24 hr later. In an initial experiment early ACs were administered prior to 25 mins of ischaemia but no preservation of renal function was observed (Figure 2A). The resulting level of functional injury was high with markedly elevated levels of plasma creatinine and thus unlikely to be responsive to any therapeutic modulation. Further studies adopted a slightly reduced level of injury (classified according to plasma creatinine). In light of work demonstrating that late ACs may exert anti-inflammatory effects [14] the influence of late ACs upon moderate kidney dysfunction was examined. However, the administration of late ACs resulted in a significant increase in plasma creatinine indicative of a worsening of kidney function (Figure 2B). This suggested that the administration of cells with significant PI positivity was detrimental and we therefore focused upon early ACs in a milder model of renal injury. Somewhat unexpectedly the administration of early ACs also resulted in a significant increase in plasma creatinine with no protection evident (Figure 2C). On the basis of these data, we did not examine the effects of early ACs in moderate injury or late ACs in mild injury. ATN was evident with widespread tubular injury in the OSOM in the ischaemic kidneys of all PBS and AC treated mice (Figure 3A). Despite the deleterious effect of early or late AC administration upon renal function in mice with mild or moderate renal IRI, the ATN scores of PBS treated and AC treated mice were comparable (Figure 3B). It is evident from the lower left quadrants of Figure 1D and E that both early and late ACs contained populations of viable non-apoptotic cells and that these cells might be responsible for the worsening of plasma creatinine. However, the administration of 20×10^6^ viable thymocytes (~95% Annexin-V^-^ PI^-^) prior to renal IRI had no significant effect upon renal function measured by plasma creatinine or ATN (Additional file 2).

**Figure 4:**
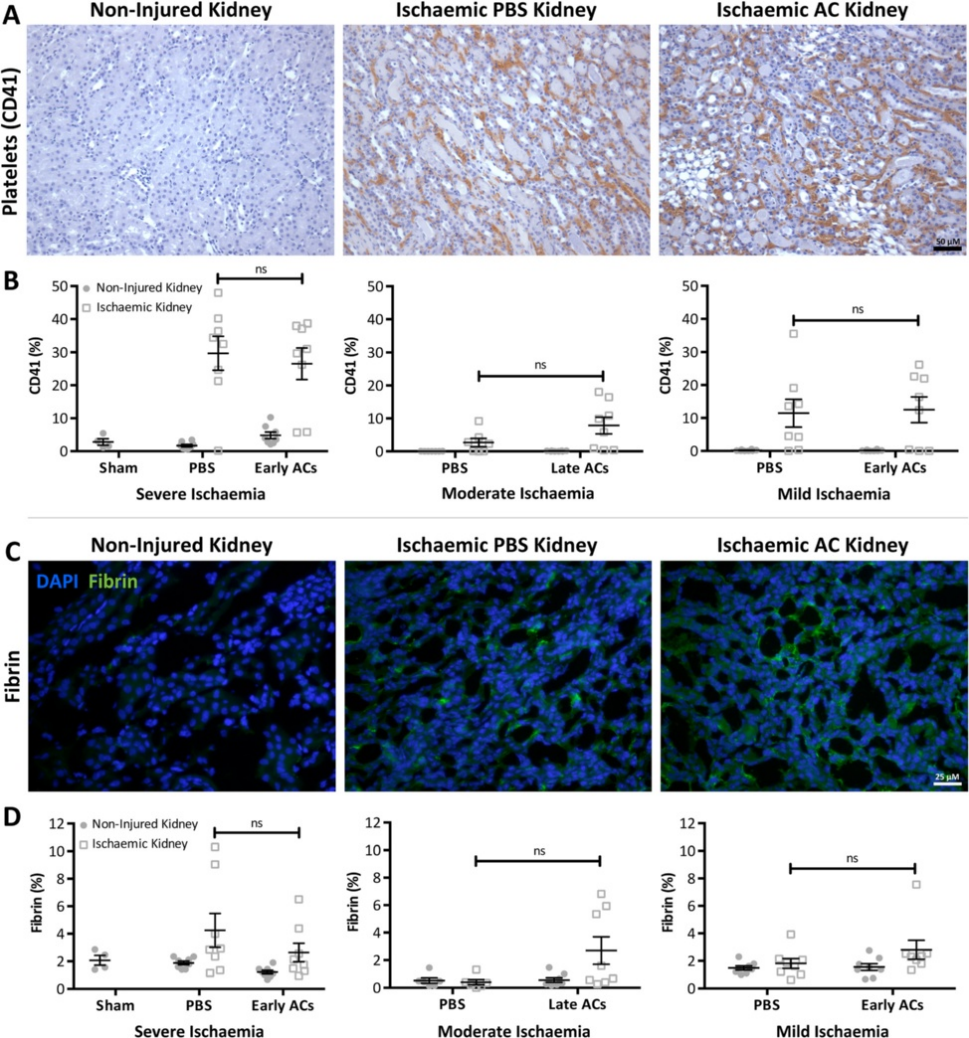
**Microvascular congestion remained unaffected following AC administration.** Renal IRI was induced in 8-week old male Balb/c mice by a right nephrectomy and ischaemia induced by occluding the left renal pedicle for 20 (mild ischaemia), 24 (moderate) or 25 mins (severe). Sham mice underwent a laparotomy only. Either PBS or 20×10^6^ early or late ACs were administered 24 hr prior to renal IRI. Mice were sacrificed 24 hr following IRI. **A & C**) Representative images of the outer stripe of the outer medulla in non-injured and ischaemic kidney sections following platelet (CD41) immunohistochemistry **(A)** and fibrin immunofluorescence **(C)** staining. In ischaemic kidneys microvascular congestion was observed as indicated by platelet and fibrin deposition. (CD41 - Magnification: ×200 & Scale Bar: 50 μM; Fibrin - Magnification: ×400 & Scale Bar: 25 μM) **B & D**) Scoring of platelet **(B)** and fibrin **(D)** staining (expressed as platelet/fibrin^+^ area) demonstrates that microvascular congestion remained similar between PBS and AC treated mice with severe, moderate and mild ischaemic injury. Grey circle symbol = Non-injured kidney Grey square symbol = Ischaemic kidney. Data expressed as mean ± SEM and analysed by two-way ANOVA. ns = non-significant. Sham (*n* = 4), PBS (*n* = 7–8), AC (*n* = 8).

The finding of unexpectedly worse renal function without an increased ATN score in AC treated mice suggested that ACs might have detrimentally reduced renal microvascular flow. The coagulation cascade is activated following ischaemic injury [17] and the microvasculature becomes congested with erythrocytes and platelet/fibrin aggregates which aggravates tissue hypoxia and limits the perfusion of residual viable and potentially functional nephrons [1,18]. ACs expressing PS are pro-coagulant and might therefore contribute to this pro-coagulant milieu [19]. To investigate this CD41^+^ platelets and fibrin were selected as markers of microvascular congestion and their deposition assessed by IHC and IF, respectively. CD41^+^ platelets and fibrin deposition was observed in the OSOM of PBS and AC treated ischaemic kidneys (Figure 4A & C), but no significant differences between AC treated and control mice were found following quantification (Figure 4B & D).

**Figure 5:**
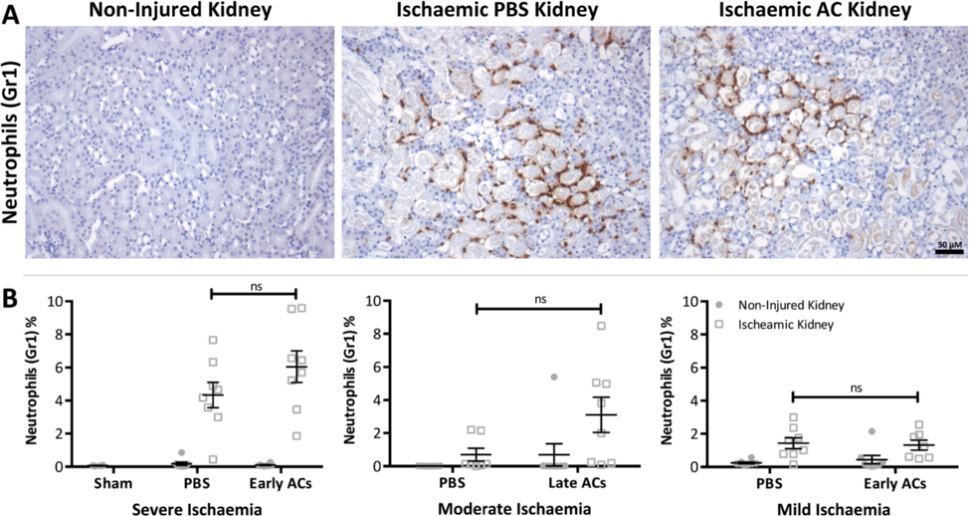
**Neutrophil infiltration occurred following ischaemia but was unaffected by AC administration.** Renal IRI was induced in 8-week old male Balb/c mice by a right nephrectomy and ischaemia induced by occluding the left renal pedicle for 20 (mild ischaemia), 24 (moderate) or 25 mins (severe). Sham mice underwent a laparotomy only. Either PBS or 20×10^6^ early or late ACs were administered 24 hr prior to renal IRI. Mice were sacrificed 24 hr following IRI. **(A)** Representative images of the outer stripe of the outer medulla in non-injured and ischaemic kidney sections following Gr1 staining. In ischaemic kidneys neutrophil infiltration was observed in areas of necrosis. (Magnification: ×200; Scale Bar: 50 μM) **(B)** Scoring of Gr1 staining (expressed as Gr1^+^ area) demonstrates that neutrophil infiltration remained similar between PBS and AC treated mice with severe, moderate and mild ischaemic injury. Grey circle symbol = Non-injured kidney Grey square symbol = Ischaemic kidney. Data expressed as mean ± SEM and analysed by two-way ANOVA. ns = non-significant. Sham (*n* = 4), PBS (*n* = 7–8), AC (*n* = 8).

With increased microvascular congestion not explaining this surprising result it was speculated that AC administration might have increased immune cell infiltration thereby elevating pro-inflammatory responses post renal IRI. Neutrophils rapidly accumulate after renal ischaemic injury [3]. They adhere to the microvascular endothelium where they may obstruct the renal microvasculature and potentially damage tubular cells by releasing reactive oxygen species [20]. To gain an insight into the inflammatory status, neutrophil infiltration was determined following IHC staining for Gr1. Neutrophil infiltration was observed in the OSOM of all ischaemic kidneys of PBS and AC treated mice (Figure 5A), however this remained equivalent in all AC treated mice and their corresponding PBS controls (Figure 5B).

### Discussion

Although AC administration has been demonstrated to reduce inflammation in diverse experimental models [10-13], the data presented in this report indicates that ACs do not protect mice from renal IRI. Whilst early ACs had no effect on severe renal IRI, the administration of early or late ACs prior to mild or moderate renal IRI respectively resulted in a further increase in plasma creatinine indicative of worse renal failure. This adverse effect was not the result of increased structural injury as ATN scores were comparable in PBS and AC treated mice.

While therapeutic interventions in the renal IRI model usually modulate both renal function and structure, functional protection alone has been observed in mice administered hemeoxygenase-1 overexpressing macrophages [21]. This protection was considered to be secondary to a dramatic reduction in CD41^+^ platelet accumulation with improved microvascular blood flow and perfusion of non-injured functioning nephrons. As plasma creatinine values were higher in AC treated mice than PBS treated controls in this study, it was hypothesized that AC treated mice might exhibit an additional worsening of microvascular blood flow post ischaemia. However, this was found to be unlikely as microvascular congestion, inferred by renal fibrin and CD41^+^ platelet IHC staining, was comparable in all PBS treated and AC treated mice.

It is surprising that early ACs aggravated renal function as early ACs have generally been regarded as anti-inflammatory in comparison to late ACs which are generally considered to be pro-inflammatory [9]. However, Gray *et al.*, found ACs at an early or advanced stage of apoptosis provided equal protection from CIA [11]. Importantly, AC-derived protection does not appear to be restricted to one cell type. The administration of between 10×10^6^ to 30×10^6^ apoptotic neutrophils [10], thymocytes [11], splenocytes [13] and human Jurkat T cells [12] have all elicited protection from inflammation in mice. In the current short report 20×10^6^ apoptotic thymocytes were administered to mice 24 hr prior to renal IRI and it is unlikely that either the AC type or number contributed to the neutral or detrimental effect on renal function.

Apoptosis and necrosis are key events in renal tubular cells following ischaemic injury [22] with inhibition of apoptosis attenuating renal inflammation [23]. However, the level of ATN did not differ between PBS and AC treated mice indicating that increased necrosis/apoptosis of renal tubular cells did not occur following ischaemic injury. Furthermore, no discernable difference in neutrophil infiltration was observed, suggesting that AC administration did not result in increased renal inflammation.

Despite previously published work demonstrating the striking ability of ACs to modulate inflammation in multiple organs [8,10-13] this data indicates that both early and late ACs were not protective in renal IRI. Indeed, depending upon the severity of ischaemic injury, administration of both early and late ACs may further impair renal function with this effect unlikely to be secondary to any contaminating viable non-apoptotic cells in the cell populations administered. These studies have not extensively probed the potential mechanisms involved in the worsening of acute kidney dysfunction by early or late ACs. ACs express PS which, in addition to being involved in the phagocytic removal of ACs, may activate the coagulation system [24]. Also, thymocytes typically undergo marked cell blebbing during the apoptotic process and it is possible that they may generate many millions of small microparticles that express PS following intravenous administration. It is thus possible that AC administration might modulate the coagulation status of recipient mice and affect the microvascular response to renal IRI. Interestingly, we did see a trend to increased platelet and fibrin deposition in mild renal IRI following administration of early ACs and moderate renal IRI following administration of late ACs but it was non-significant. Early ACs contained some PI positive cells that have lost membrane integrity whilst the majority of late ACs were PI positive. Although we did not undertake a direct head-to-head comparison of the effects of early and late ACs, it is possible that the adverse effects of late ACs might involve the release of intracellular damage-associated molecular pattern molecules such as high-mobility group box chromosomal protein 1 that may activate cells via Toll-like receptor 4 [25]. This work contrasts with the AC-derived protection observed in other organs and suggests that AC administration does not have translational potential for patients with AKI secondary to renal hypoperfusion.

## Additional files

Additional file 1:
**Isotype controls for Gr1 and CD41 were negative on non-injured and ischaemic kidney.** Renal IRI was induced in 8-week old male Balb/c mice by a right nephrectomy and ischaemia induced by occluding the left renal pedicle for 24 mins. Representative images of the outer stripe of the outer medulla in non-injured and ischaemic kidney sections following staining with isotype control antibodies for CD41 and Gr1. Both isotype control antibodies exhibit no staining in non-injured and ischaemic kidneys. (Magnification: ×200; Scale Bar: 50 μM). Additional file 2:
**Administration of viable thymocytes prior to renal IRI had no influence on renal function or ATN.** Dissociated thymocytes were prepared from 4-week old male Balb/c mice. Cell viability was assessed by Annexin-V and Propidium Iodide (PI) staining and flow cytometry. Either PBS or 20×10^6^ viable non-apoptotic thymocytes were administered to 8-week old male Balb/c mice 24 hr prior to renal IRI induced by a right nephrectomy and occlusion of the left renal pedicle for 20 mins. Mice were sacrificed 24 hr following IRI. **A)** Viable thymocytes were approximately 94% Annexin-V^-^ PI^-^. **B)** Administration of viable thymocytes prior to renal IRI had no significant influence on renal function measured by plasma creatinine. **C)** Scoring of ATN (acute tubular necrosis) (expressed as the percentage of necrotic tubules in the outer stripe of the outer medulla) demonstrates that the structural injury remained similar between mice that received PBS or viable thymocytes prior to renal IRI. Grey circle symbol = Non-injured kidney Grey square symbol = Ischaemic kidney. Data expressed as mean ± SEM and analysed by either student’s *t*-test or one-way ANOVA. ns = non-significant. PBS (*n* = 5), Viable thymocytes (*n* = 5).

## Abbreviations

AC(s): Apoptotic cell(s)
AKI: Acute kidney injury
ATN: Acute tubular necrosis
CIA: Collagen induced arthritis
IF: Immunofluorescence
IHC: Immunohistochemistry
IL: Interleukin
IRI: Ischaemia reperfusion injury
LPS: Lipopolysaccharide
OSOM: Outer stripe of the outer medulla
PI: Propidium iodide
PS: Phosphatidylserine
TGF-β: Transforming growth factor-β
TNF-α: Tumour necrosis factor-α
