# Supplementary figures and images for: Apoptotic cell administration is detrimental in murine renal ischaemia reperfusion injury
Source: J Inflamm (Lond). 2014 Oct 10;11:31. doi: 10.1186/s12950-014-0031-6 (PMC4195900; doi:10.1186/s12950-014-0031-6)

**Non-Injured Kidney**

**Ischaemic Kidney**

**Gr1 Isotype**

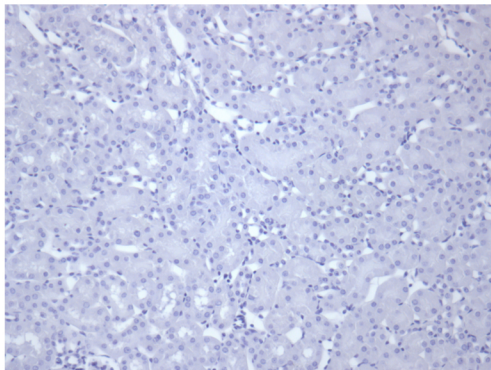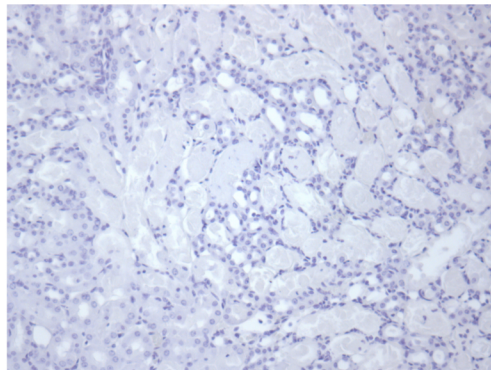

**CD41 Isotype**

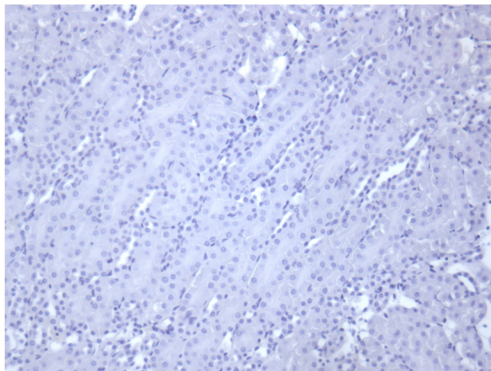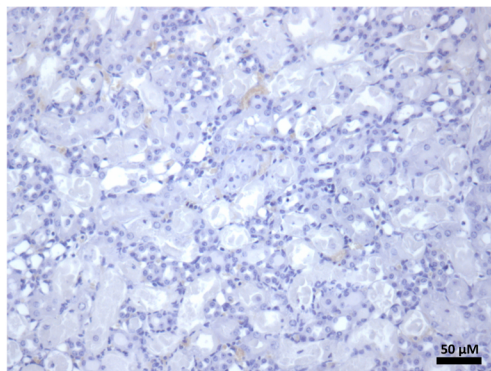

Supplement: Additional file 1: — Isotype controls for Gr1 and CD41 were negative on non-injured and ischaemic kidney. Renal IRI was induced in 8-week old male Balb/c mice by a right nephrectomy and ischaemia induced by occluding the left renal pedicle for 24 mins. Representative images of the outer stripe of the outer medulla in non-injured and ischaemic kidney sections following staining with isotype control antibodies for CD41 and Gr1. Both isotype control antibodies exhibit no staining in non-injured and ischaemic kidneys. (Magnification: ×200; Scale Bar: 50 μM). [file 12950_2014_31_MOESM1_ESM.pdf]

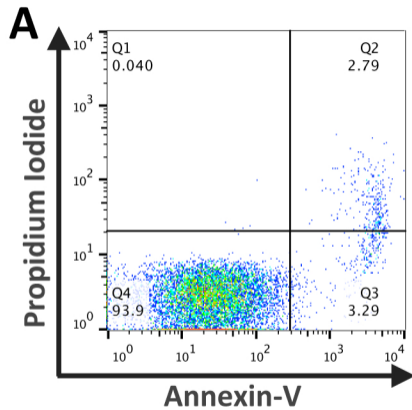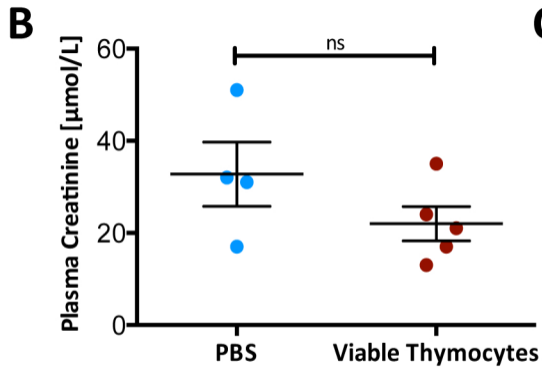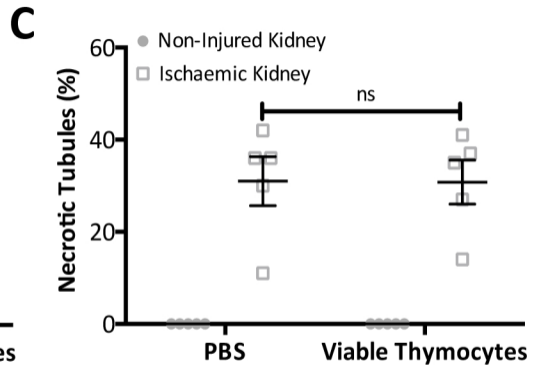

Supplement: Additional file 2: — Administration of viable thymocytes prior to renal IRI had no influence on renal function or ATN. Dissociated thymocytes were prepared from 4-week old male Balb/c mice. Cell viability was assessed by Annexin-V and Propidium Iodide (PI) staining and flow cytometry. Either PBS or 20×106 viable non-apoptotic thymocytes were administered to 8-week old male Balb/c mice 24 hr prior to renal IRI induced by a right nephrectomy and occlusion of the left renal pedicle for 20 mins. Mice were sacrificed 24 hr following IRI. A) Viable thymocytes were approximately 94% Annexin-V- PI-. B) Administration of viable thymocytes prior to renal IRI had no significant influence on renal function measured by plasma creatinine. C) Scoring of ATN (acute tubular necrosis) (expressed as the percentage of necrotic tubules in the outer stripe of the outer medulla) demonstrates that the structural injury remained similar between mice that received PBS or viable thymocytes prior to renal IRI. Grey circle symbol = Non-injured kidney Grey square symbol = Ischaemic kidney. Data expressed as mean ± SEM and analysed by either student’s t-test or one-way ANOVA. ns = non-significant. PBS (n = 5), Viable thymocytes (n = 5). [file 12950_2014_31_MOESM2_ESM.pdf]
